# The complete mitochondrial genome of *Chrysolina aeruginosa* Fald (Coleoptera: Chrysomelidae)

**DOI:** 10.1080/23802359.2020.1832935

**Published:** 2020-11-09

**Authors:** Wei-Wei Xie, Jia-Yi Ma, Huan He, Chen-Yan Huang, Guang-Hong Liang, Fei-Ping Zhang, Hui Chen

**Affiliations:** a College of Forestry, Fujian Agriculture and Forestry University, Fuzhou, China; b Key Laboratory of Integrated Pest Management in Ecological Forests, Fujian Province University, Fujian Agriculture and Forestry University, Fuzhou, China

**Keywords:** Mitogenome, *Chrysolina aeruginosa*, phylogeny, Chrysomelidae

## Abstract

In this study, the first complete mitochondrial genome of *Chrysolina aeruginosa* Fald was assembled and analyzed. The total length of this mitochondrial genome is 16,335 base pairs. It consists of 13 protein-coding genes, 22 transfer RNAs, two ribosomal RNAs, and an AT-rich region. Phylogenomic analysis indicated that *C. aeruginosa* Fald is sister to *Chrysodinopsis* sp. This study provides new molecular data for the further taxonomic and phylogenetic studies of the Chrysomelidae of Coleoptera.

*Chrysolina aeruginosa* Fald is a major pest of *Artemisia ordosica* Krasch. In recent years, this phytophagous beetle has spread rapidly throughout northwest China, leading to mass mortalities of *A. ordosica* Krasch, which has produced great damage to the local ecology (Da-Zhi et al. 2014). In the present study, the complete mitochondrial genome of *C. aeruginosa* Fald is reported contributing for better understanding its evolution and population genetics, and also providing significant information for the phylogeny of Chrysomelidae.

The samples of *C. aeruginosa* Fald were caught by the traps of attractive substance from Minhou, Fujian Province, China (119°4′35″E, 26°14′9″N). All voucher specimens were assigned with a unique code and deposited in the Key Laboratory of Integrated Pest Management in Ecological Forests, Fujian Province University, Fujian Agriculture and Forestry University (voucher no.YJ-202006). The genomic DNA was extracted using a phenolchloroform extraction protocol from the legs of samples (Russell and Sambrook 2001). DNA quality and concentration were determined using Nanodrop (Thermo Fisher Scientific,Waltham, MA, USA). Then, the DNA library with fragments of 300 bp in size was constructed by PCR amplification and the size selection by Agencourt AMPure XP-PCR Purification Beads (Beckman Coulter, CA, USA) and Agencourt SPRIselect Beads (Beckman Coulter, CA, USA). The multiple samples were mixed and sequenced using Illumina Hiseq 2500 (Genesky Biotechnologies Inc. Shanghai, China).

A total of 46,513,652 clean reads were obtained by filtration from the 47,161,712 raw reads. After the *de novo* assembly of metaSPAdes (Nurk et al. 2017), the complete circular mitogenome was obtained. The total length of this mitochondrial genome is 16,335 base pairs (GenBank accession No: MT826861). Three characteristics of protein-coding sequence, tRNA, and rRNA were obtained by the annotated result of mitoMaker (Bernt et al. 2013). And, tRNA genes were predicted by using tRNAscan (Lowe and Eddy 1997). A total of 37 genes were annotated, which contained 13 protein-coding genes, 22 transfer RNA (tRNA), and two ribosomal RNA (rRNA), there was also an AT-rich region. To confirm the phylogeny of *C. aeruginosa* Fald, 13 complete genomes of different species of Chrysomelidae were obtained from GenBank, and aligned using HomBlocks software (Bi et al.^2018^). A Maximum Likelihood (ML) tree with 1000 bootstrap replicates was inferred using MEGA 7.0 (Sudhir et al. 2016). The result of ML phylogenetic tree showed that *C. aeruginosa* Fald is closely related to *Chrysodinopsis* sp. EMHAU-1507081(Figure 1). The mitochondrial genome of *C. aeruginosa* Fald will provide useful genetic information for further study on genetic diversity and genetic evolution of Chrysomelidae species.

**Figure 1.**
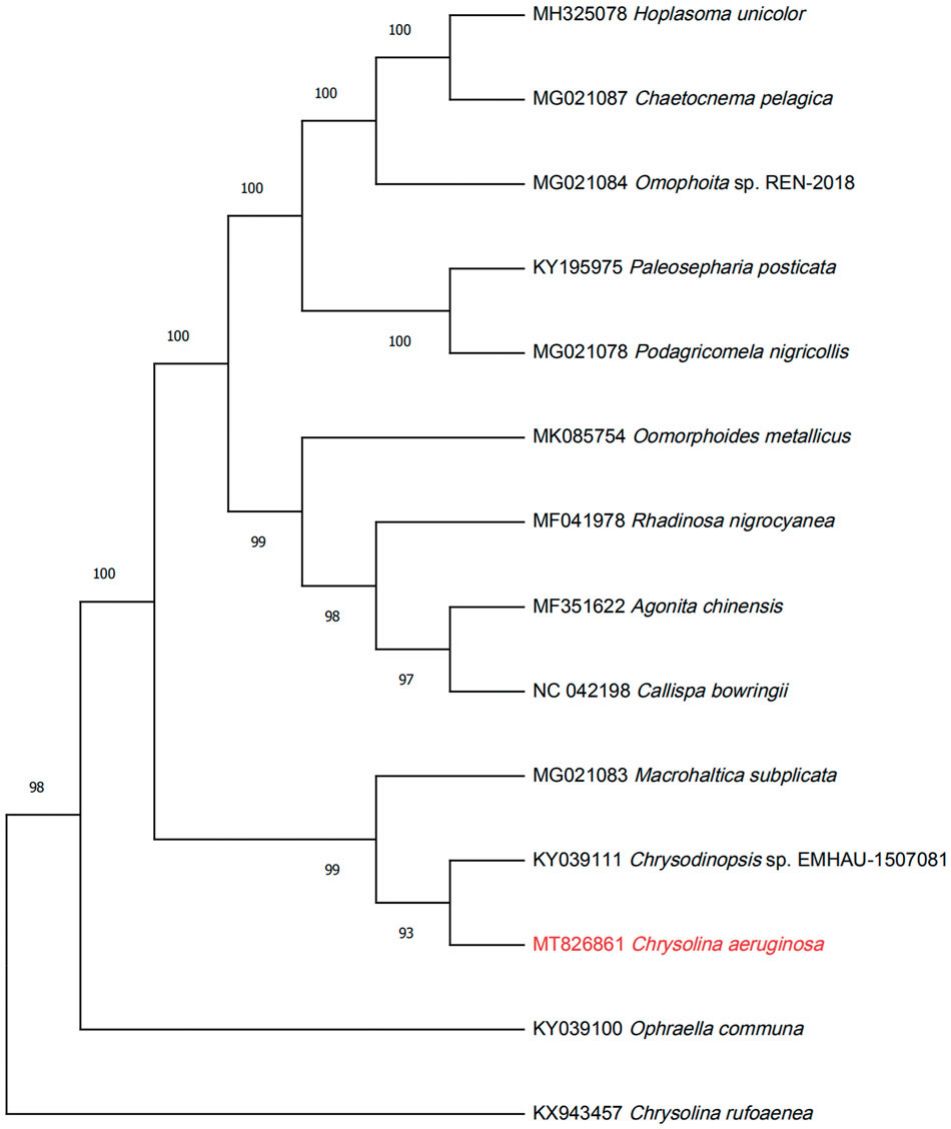
The Maximum Likelihood (ML) phylogenetic tree based on 14 mitochondrial genome of Chrysomelidae. Values along branches correspond to ML bootstrap percentages.

## Data Availability

The data that support the findings of this study are openly available in”NCBI”at https://www.ncbi.nlm.nih.gov/, reference number MT826861.
